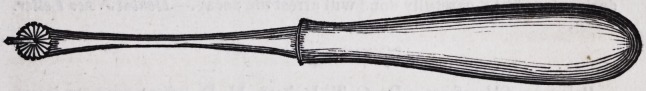# Quarterly Summary

**Published:** 1853-04

**Authors:** 


### QUARTERLY SUMMARY.

*Caries of the Teeth.*—The January number of the Dental Register of the West, for 1853, contains the following interrogatories, propounded to the Mississippi Valley Association of Dental Surgeons, in 1846, by the late Professor Drake.

1st. What is the nature of that diathesis, or constitutional predisposition or disorder (if any,) which so often occasions decay in the deciduous teeth of our children?

2nd. To what causes, external or pathological, local or constitutional, shall we ascribe the premature decay of the second teeth, in the west?

Is an hereditary, scrofulous diatheses a cause of infirm teeth?

Is dyspepsia a cause of early decay?

Does the acid thrown up by the dyspeptics, in paroxysms of that disease, act chemically on the teeth?

What is the effect of repeated salivation on the teeth and gums?

What are the effects of tobacco on the teeth, and are those of chewing and smoking the same?

3d. Has the tartar of the teeth a constitutional origin?

4th. Is the decay of the teeth greater in the west than in the Atlantic States, in the same latitudes; and is there any difference in different latitudes, in the same meridians?

5th. Is there any difference as to soundness of teeth, between our native and foreign population?

6th. Are the teeth of our colored people less subject to decay than those of white, who labor and live in a similar manner, as to diet and drinks?

Professor Taylor, chairman of the committee to whom the foregoing questions were referred, published in the last (January) number of the Dental Register, a reply to the first interrogatory, promising a continuation of the report.

In replying to this question, Prof. Taylor assumes that the premature decay of the deciduous teeth is attributable to deficiency of earthy salts in the organs, arising from too great serosity of the blood occasioned by feeble or impaired health of the mother during the intra-uterine existence of the child and the period of lactation. This condition of the blood as well as the state of the health of the mother, the Dr. shows, by a lengthy argument and numerous facts, to be owing to temperament, habit of body and the kind and quality of diet used during the development and ossification of the teeth, by which the functions of the formative tissue of these organs are influenced. Lymphatico-serous, or mucous dispositions, and especially when attended with a scrofulous diathesis, he regards as unfavorable to the production of teeth of a sufficiently dense structure to resist the action of the causes of decay to which all teeth are, more or less exposed.

Many of the views of Dr. T. accord with those expressed by the senior Editor in a paper published about twelve years ago in one of the early numbers of the Journal, explanatory of the differences in the liability of the teeth of different individuals, and even in the same mouth, to decay; and subsequently embodied in a chapter in part second of his Principles and Practice of Dental Surgery.

*Filling Teeth.*—Dr. Clark recommends in the Dental Register the use of gold foil made into cylinders for filling teeth, which he introduces into the cavity parallel to its walls until it is full. The cylinders are made by rolling a strip of gold previously folded to a width corresponding to the length required for the cylinder on a watchmaker's pivot-broach, broken off an eighth of an inch from the shoulder, and this part having been first reduced to a point. These cylinders are introduced with Dr. Taylor's forceps or tweezers. After the cavity is apparently filled, a round pointed instrument is forced between the cylinders and others of a smaller size inserted, after having filled, we presume, the opening previously made, until the cavity is completely and thoroughly filled. The gold having been thoroughly consolidated, the surface of the filling is finished by means of cutting and polishing substances instead of the file and burnisher as employed by some dentists. The idea of using gold in cylinders for filling teeth, Dr. C. says he obtained from Dr. F. H. Badger, who has enjoyed a most enviable reputation for skill in this operation for more than twenty years, in Tennessee.

For preparing and filling roots of teeth, Dr. C. employs the finest watchmaker's broaches. Those used for the removal of the pulp have barbs cut on them, by means of which it is easily removed. Several sizes are required for the introduction of the gold. The foil is introduced by a strip wound upon one of these broaches to the very point, previously made perfectly smooth and untempered. Thus armed, the instrument is introduced into the fang; the gold is now lightly grasped with a pair of tweezers and the broach withdrawn, leaving the gold in the root. A piece of whalebone scraped down to the proper size is thrust up by the side of the gold to make room for another piece, and thus piece after piece is introduced until the cavity in the root is filled. Dr. C. acknowledges to have obtained this method of filling roots of teeth also from Dr. Badger.

*Springing of Plates.*—Dr. J. Harris expresses the opinion in the Dental Register that the springing of plates in soldering teeth to them is chiefly owing to the unequal application of heat, the peripheral edge and the teeth being covered with plaster and sand prevents it from acquiring as high a degree of temperature as the central part upon which the flame of the lamp is projected. The unequal expansion thus produced may cause a thickening of the central portion of the plate, or an increase of its convexity, or both, so that when the piece cools the outer or peripheral portion is liable to be drawn inwards.

To prevent this, heat the central portion of the plate to as high a temperature as will be required for soldering, with the peripheral part protected previously to swaging it the last time. In preparing the piece for soldering, use as much sand as plaster, as in this case the mixture will expand as much as the plate in heating, and on cooling will bring back the peripheral portion of the plate to its original position.

---

*Extraction of Teeth with the Key.*—The Key instrument being still employed by some practitioners, Dr. J. Taylor, (see Dental Register, Jan'y. No. 1853,) after describing the kind of key which he thinks best adapted to the operation, recommends in the extraction of a lower molar that the fulcrum, wrapped with the corner of a fine napkin or silk handkerchief, as a general rule, be placed on the lingual side of the tooth, as in this case, the lower molars inclining a little inwards, the force applied for the ex traction of the tooth is exerted more nearly in the direction of the root than it would be if the fulcrum were applied externally on the buccal side. The point of the hook should be placed as low down on the neck of the tooth as possible.

With regard to the position of the operator, the Dr. says he stands to the right and a little before the patient, though sometimes but not often to the left, the head of the patient should be nearly upright. For the removal of a tooth on the right side, he employs as a general rule, the right hand, and for the left side the left, with the palm in either case on the upper side of the handle of the instrument. With the other hand the jaw is supported and the hook of the instrument held firmly upon the tooth. With the key thus adjusted the requisite amount of rotatory force is applied for the dislodgment of the organ. Dr. T. employs a smaller key and hook for the removal of the bicuspids.

In the extraction of an upper molar "we have the head thrown far back, and stand on a stool at the back of our patient," so that the tooth to be extracted can be seen, holding the instrument in the right or left hand as most convenient, and the hook with the forefinger of the other, applying the fulcrum as a general rule on the palatine side of the tooth.

When the tooth is situated on the outside of the circle, whether in the upper or lower jaw, as is sometimes the case, or when the buccal side of the crown is so much decayed as not to afford a sufficiently secure support for the point of the hook, the fulcrum may be applied on the outside, and the hook on the inner side of the tooth.

*The Teeth as Objects of Study.*—In an article under the above title, (Southern Journal of Medical and Physical Sciences, Jan'y. 1853,) Dr. B. Wood says:

The celebrated John Hunter, who is charged with having propagated the erroneous doctrine, that the teeth are inorganic, and who certainly did regard them as "extraneous bodies with respect to a circulation through their substance," was nevertheless well aware of the nature of their relations. He urges attention to the teeth "not only for the preservation of themselves," but "also on account of other parts with which they are connected; for diseases of the teeth," he continues, "are apt to produce diseases in the neighboring parts, frequently of very serious consequences." * * *. "Their diseases, considered abstractly, are indeed very simple, but by the relations which the teeth bear to the body in general, and the parts with which they are immediately connected, they become extremely complicated."*

M. Jourdain, of France, who was cotemporary with John Hunter, insists in his writings upon the importance of these connections, and his work on the *Surgery of the Mouth* is replete with cases in illustration of it. He illustrates the pathological relations of the teeth with the antrum of Highmore, the tongue, palate, maxilla, and salivary glands, with the eyes, the ear, &c., with the stomach, intestines, uterus, brain, and general system. And he draws a strong line of discrimination between true dental surgery and what he terms the "*mechanism of dentistry,*" urges the attention of surgeons to the former, and ends his work by saying: "We must be faithful and diligent in the study of this essential branch of the healing art—the Surgery of the Mouth."†

The sympathy of the general system with the teeth, in a diseased condition, has subsequently attracted the special notice of medical writers, and none perhaps have more forcibly illustrated it than Dr. Rush, who traced many cases of disease to the bad condition of the teeth, such as dyspepsia, neuralgia, epilepsy, rheumatism, fever, &c.

The teeth themselves, however, were formerly regarded as comparatively unimportant, and their diseases, "considered abstractly," as very simple. Hence their study and treatment were neglected by the profession, and the care of them fell into the hands of whomsoever saw fit to assume this responsibility. So long as the dentist confined himself to the teeth, little danger was apprehended; though he was often admonished against transcending his limits or rebuked for doing so. John Hunter, in speaking of abscess of the jaws, says: "In such cases, it is but too common for the dentist to be very busy, and perhaps do mischief through ignorance." But dental surgery, in its comprehensive sense, had not as yet been abandoned by the medical profession.

Thus it appears that the great error which physiologists of former times

* Natural History of the Teeth.

† Diseases and Surgical Operations of the Mouth, translated by Dr. P. H. Austen.

fell into, consisted, not in a lack of knowledge concerning the connections of the dental system, but in a misconception as to the nature of the teeth themselves; an error which has continued prevalent down to the present time. Every observant physician is well aware of the important sympathies of the dental system, while at the same time the teeth seem to be generally regarded by the profession as of little consequence, either as objects of pathological or physiological investigation. And to this latter cause, aided by others, we think is to be traced the decline of dental surgery and its almost entire separation from medicine. But the researches of modern times have discovered in the teeth a structure highly organized and remarkably endowed with vitality. The extent of their sympathies is also better understood, easier accounted for, and more fully appreciated; hence the recent revival of dental surgery as a medical science, and the consequent interest which it should elicit from the whole profession.

Professor Horner, in his *Anatomy and Histology*, says:

"Those untractable and mineral-like bodies, the teeth—exciting once almost the doubt of intrinsic organization, now penetrated by the microscope in a wonderful manner, and exhibiting the most surprising organization; an organization so characteristic and permanent, that it has become one of the most efficient means of discriminating in fragments of animals the kind to which they belonged, whether of the present or a former order of the world. Each of the component parts of the teeth, the cement, enamel and ivory, exhibiting a specific organization; its fibrils and its tubules, whose arrangement, in being specific, gives decided character to the specimen in question."

The zoological and paleontological importance of the teeth consequently rises to the first magnitude; affording, as they do, the principal basis, in many respects, of a natural or physiological classification of existing animals, and the only criterion for classifying many extinct species; contributing remarkably to modify, enlarge and perfect this department of science, and leading to the discovery of lost "links," and hence to the reunion of sundered parts, in the chain of being hitherto regarded as irreparable or even originally defective.

Hugh Miller gives a graphic sketch, in his inimitable style, of a species of the ancient Celacanth family, which partakes both of the ichthyic and and reptilian character, and which, "for untold ages, has had no living representative." "The jaws were thickly set with an outer range of true fish teeth, and more thinly with an inner range of what seem *reptile* teeth, that stood up, tall and bulky, behind the others, like officers on horse back, seen over the heads of their foot soldiers in front." In another place, in speaking of a member of the same family, the *Asterolepis,* wherein "immediately behind the front row—in which the teeth present the ordinary ichthyic appearance—there was a thickly set row of huge *reptile* teeth;" he remarks: "The reptile had, as yet, no existence in creation; but we see its future coming symbolized in the dentition of this ancient ganoid; it, as it were, shows the *crocodile* lying entrenched behind the fish."*

Professor Owen, in his elaborate *Comparative Anatomy of the Teeth,* commenting on the recently discovered *Rhynchosaurus* as a connecting link between the classes Reptila and Mammalia, exclaims: "Our interest rises almost to astonishment, when, in a saurian skull, we find superadded to the horn-clad mandibles of the tortoise, a pair of tusks, borrowed, as it were, from the mammalian class, or rather foreshadowing a

*** Foot Prints of the Creator, or the Asterolepis of Stromness.**

structure, which in the actual creation, is peculiar to certain members of the highest organized warm-blooded animals."

The hard parts of the body are nearly all the relics we possess of a former fauna; and as the teeth are, in many instances, the only durable records of the past, remaining perfect in cases where the bones themselves have mouldered away, it is most fortunate to science that they should present characters so significant—serving not only by their form and relative position, but even by their structure, to designate the place, form and habits of different species and genera.

"So constantly do they correspond," says Professor Agassiz, in his Principles of Zoology, "with the structure of other parts of the body, that a single molar is sufficient to indicate not only the mode of life of the animal to which it belongs, and show whether it feeds on flesh or vegetables, or both, but also to determine the particular group to which it is related." "Even the internal structure of the teeth is so peculiar in each group of animals, and yet subject to such invariable rules, that it is possible to determine with precision, the general structure of an animal, merely by investigating the fragment of a tooth under a microscope."

Nor is this all. Comparative dental *Physiology* opens new wonders to the gaze of the naturalist, and pours an additional flood of light upon his science. The formation, development, and decidence of the teeth furnish zoological indications as remarkable as those afforded by form, position and structure; thereby greatly extending our knowledge respecting the animal kingdom, its natural divisions, and the correct principles of classification.

Such revelations, so important in their bearings upon comparative and general anatomy, and consequently upon numan physiology—a science inseparably allied to the former—should not fail to elicit the attention of the medical profession. The human teeth especially, solicit investigation, not only as the standard of comparison in odontological researches, but as affording valuable indications of the structure and functions of the human system; such investigation, while replete in itself with interest and profit, greatly contributing to the elucidation of other subjects of physiological and pathological inquiry. In point of structure the teeth furnish invaluable aid to a correct knowledge of the other hard parts. Dr. Carpenter speaking of the tissues of teeth and bone says, "the structure of both of them is well adapted to demonstrate the distinction between the tissues themselves, and those subsidiary parts, by which they are connected with the rest of the structure." The same might be said with respect to the pathological conditions of both.

It is a curious fact, that a tooth comprises within itself nearly every tissue of the body, from the least organized to the most highly vitalized. Commencing within, and proceeding outward through the natural opening at the apex of the root, we have the pulp, comprising the nerve and blood vessels with their tunics, then the peridental membrane investing the root and giving off a duplicature to the periosteum of the alveolus—which is properly a part of the tooth since it grows with it and wastes away when it is removed—next, in juxtaposition with this osseous structure is the cementum, nearly allied to true bone, then the compact but still highly organized dentine, or ivory, and lastly the enamel or earthy covering, which may be regarded as inorganic. Or commencing without, we pass from a crystalline mineral substance, along a structure of high and still higher organization, to that which is all vitality, all sensibility, the vehicle of impressions to the brain, and through this, of sensations to the mind. Thus affording an instance of a blending and union of organic with inorganic structure, a regular transition from material to immaterial nature, a continuous link between mind and matter.

The germination, ossification, development, decidence and succession of the teeth, present one of the most beautiful and wonderful series of phenomena in the whole range of physiology. Nor are these without instruction in regard to other vital phenomena. We find in them, taken individually, a repetition of analogous processes throughout the general system, and, collectively considered, a magnified representation of the evolution of the cell-germ upon the one hand, and upon the other, a miniature imitation of embryonic development.

But the vital characteristics of the teeth, as yet but little investigated by medical inquirers in general, certainly much better understood by those who have made this part of the body a subject of practical as well as theoretical acquainaance, present peculiarities and indications equally remarkable with those already referred to. We do not now allude to the connections of the teeth, whether through their nervous and vascular tissues with the general system or with any of its organs, or through the peridental membrane with the periosteum, bone and muscles of the neighboring parts; these are indeed worthy of attentive study in consequence of important functions and sympathies; but we here refer to the characteristics of the true dental tissue, in its normal condition in the living economy.

Apart from its remarkable organization and mode of formation, the dental tissue, or tooth bone, is most certainly endowed—whatever may be inculcated in medical works to the contrary—with all the essential characteristics of vitality—characteristics which, though perhaps beyond the cognizance of theoretical physiologists, are nevertheless sufficiently obvious to every observant dental surgeon. This tissue has within itself a system of circulation by which and through which it receives nutrition from the general circulation. It is subject to a grade of inflammatory action, admits of interstitial absorption and disposition to a certain extent, and is capable of, and liable to, accessions and waste of substance, from without and within, as the result both of physiological and pathological processes. It is supplied undoubtedly with nervous fibrils, by means of which it is possessed of irritability, and of sensibility to such an extent as to render the teeth subservient to the purposes of general sensation, and, to some extent, organs of special sensation. In consequence of this they are susceptible, often keenly so, to external impressions as from themral, electrical, and chemical changes, and even from ordinary tactility. This susceptibility is often greatly heightened by inflammatory action, and may become so exalted as to render the exposed surface of the dentine as intolerant of irritants as the dental pulp itself, requiring for its subjugation a course of local treatment preparatory to the operations that may be demanded in such cases.

*Filing the Teeth.*—Among the objects for which the file is used in operations on the teeth, are stated by Dr. B. Wood, (Southern Journal of the Medical and Physical Sciences, March, 1853,) to be, first, the removal of caries in its incipient stages; second, the separation of teeth preparatory to filling, and lastly, for reducing the length of teeth that are longer than the adjacent organs; also, for smoothing teeth which have become roughened from wear or fracture. The use of the file for separating teeth is only admissible in cases of actual decay, and even then it should be avoided in young subjects if possible. But when it is done, the separation should be made in such a manner as to prevent the approximation of the filed surfaces. After filing, the surfaces should be made smooth and polished, and when the operation is performed preparatory to filling, the space should be made wide enough to permit the operation to be performed in the most perfect manner, making the separation wide behind, when made between the front teeth, and narrow in front, so that the appearance of the teeth may not be injured.

*A "New Form" of Arsenic for destroying Dental Nerves and Obtunding the Sensibility of Dentine.*—Dr. B. Wood recommends in the Southern Journal of Medical and Physical Sciences, the metallic form of arsenic obtained from the shops under the name of fly-stone, as possessing advantages for the purposes above mentioned, over the other preparations of this powerful agent. It is finely pulverized and applied in the usual way for destroying nerves in teeth.

*Hemorrhage from the Gums.*—Mr. Bullock reports in the London Lancet, a case of hemorrhage of the gums of a delicate looking girl, nine years of age, successfully treated by Mr. Ure, by the internal use and direct application of spirits of turpentine; after tannin, nitrate of silver and applications of ice had been used without any good effect.

*Hemorrhage from the Extraction of Teeth.*—Dr. Wm. R. Webster reports in the Dental News Letter, January, 1853, a case of hemorrhage from the extraction of a tooth in which he employed successfully, sulp. magnesia, administered in sufficient quantity to operate mildly on the bowels, and says, it has never failed in his hands to arrest the bleeding even in those cases in which there is a hemorrhagic diathesis.

*Death from Hemorrhage, consequent upon Lancing the Gums.*—Mr. Taynston reports a case in the London Lancet of a little patient six months old, who had the gums lanced on Sunday and in spite of various styptics, including the actual cautery, died from the loss of blood on Tuesday following.

Mr. Bouney on the same, goes on to say:—The first point to be considered, is how the occurrence may be prevented. If a hemorrhagic diathesis exists, no one knowing this, would think of scarification, therefore it may be advisable to inquire whether that peculiar constitution exists in the family.

Another most important rule is to make no long incision, but if several teeth are advancing on the same line, let the scarifications be short and detached.

Mr. B. further says, that he has nearly always succeeded in getting it under by pressure with the finger on a suitable compress saturated with a strong solution of nitrate of silver, and notices the case of Capt. V——, where he was partially successful. Discovering the jet of a small artery, he touched it with a point of lunar caustic and had no further trouble. He would however avoid the use of solid nitrate of silver with infants, on account of the delicacy of the mucous membrane, especially with those suspected of hemorrhagic diathesis.

He would deem it prudent to give from time to time a few drops of the tincture of ergot or other internal styptic (perhaps the tincture of matico) so long as hemorrhage continued, and to attend immediately, on learning that a child's gums were bleeding unusually after scarification.

*Inserting Pivot Teeth.* By A. T. Willard, D. D. S.—My plan of setting, after excising and drilling the fang in the usual way, is to fit the fang with an instrument I call the counter-drill. I cut the fang to the desired depth, to hide the base of the artificial crown below the gum, taking care not to disturb the periosteal membrane more than can be avoided. I then, with a small sized counter-drill, cut down the fang about a sixteenth of an inch, leaving a peripheral to support the gum in its natural shape. Fit the artificial crown to the last used counter-drill and if the work has been well designed and executed, the crown must perfectly fit the fang. See cut for counter-drill.

I use five different kinds of this instrument. The centre pivot should be a trifle smaller that the pivot hole, and long enough to guide the instrument to cut perfectly at right angles with the pivot hole. To fit the crown to the last used counter-drill, pass the centre pivot into the socket of the crown and fit it by grinding, &c. The instrument should have a thin copper follower to protect the cutting edge from the tooth.

I use the pure tears of mastic, dissolved in chloroform to coat the wood pivot before forcing it home to the fang to make the crown and fang imperforable to the fluid of the mouth. To make pivot wood, use a steel plate about an eighth of an inch thick, with holes of sizes to suit for pivots, with a projecting cutting lip upon one side; the holes should be slightly narrowing through, so that by pressing the wood and turning it into this plate, the pivot would come through, finished for use, round, smooth, and compressed.—*Dental News Letter.*

The above method was described to the senior editor in 1831 by Dr. Lee, dentist, formerly of Alabama.—Eds.

*Removing Teeth from Plates.* By A. B. Child, of Boston.—Imbed the set, from which the teeth are to be removed, in plaster and sand, the same as for soldering; with a pencil brush spread a thin coat of fine quartz or sand, mixed with a little plaster and water, over the whole surface of the plate, coming to the edge where the solder which holds the teeth to the plates terminates, leaving exposed the gold on the backs and the entire backs of the teeth. The plaster and sand in which the teeth are imbedded should be cut away in a small point to give room for a wire to be applied upon each tooth, just in front of its cutting edge. Heat the set gradually in a charcoal fire, taking care that the bottom of the soldering pan is covered with a bed of fine coal, on which the teeth may fall. Use a blow pipe to fuse the solder, while an assistant, with a long wire, tips the teeth inward, one by one till they are all removed.—*Dental News Letter.*

*Treatment of Erosion of the Enamel.*—R. Brooks, dentist, says, cleanse the cavity perfectly, take a small piece of nitrate of silver, place it in the cavity, dip an instrument in clean water, place it (with the little water attached) upon the nitrate of silver and move it a little till the silver is dissolved, being careful to have it enter all the parts; this turns the cavity a dark color; but if carefully done will arrest the decay.—*Dental News Letter.*

*Poisonous Chloroform.*—Dr. C. T. Jackson, M. D., after numerous investigations, thinks the numerous deaths which have taken place recently from the use of chloroform, must have been produced by the presence of some poisonous compound of amyle, the hypothetical radical of fusel oil, or the oil of whisky, and states the following facts and inductions.

1st. When chloroform and chloric ether was made from pure alcohol diluted with water, no fatal accidents took place from its judicious administration.

2d. Chloroform made from common corn, rye and potato whisky caused frequent deaths, even when the utmost care was taken in its administration.

3d. A sudden death occurred in Chelsea, where this kind of chloroform was probably contained in the alcoholic solution, and the post mortem appearance of the subject indicated the usual effect of poisoning by chloroform.

He says it may justly be inferred that some poisonous matter exists in the cheap chloroform of commerce, and advises that physicians and surgeons should return to the use of pure sulphuric ether (oxide of ethyle,) as originally prescribed by him.

He has compounded chloroform, say about one-fourth or fifth part, with sulphuric ether and extensively used this preparation in the production of anæsthesia, always taking care that his chloroform was pure.

To obtain this poison, spoken of above, he mixed chloroform with hyperchlorite of lime (bleaching powders) and water in a retort, the same way alcohol is prepared for the distillation of chloroform. After some hours the retort was placed in a water bath and distillation effected, a clear colorless liquid coming over, having the peculiar *odor of bad chloroform.* He again mixed the product with new bleaching powder and water, and after three hours with frequent agitations, gave what he called pure unmixed poison.

It is important to know, he says, now that this fusel oil has been introduced as a remedy in phthisis, that when it is inhaled it may produce fatal results.—*Boston Med. & Surgical Journal.*

In a letter from Dr. N. C. Perkins to C. T. Jackson, M. D., on the subject of fusel oil, the writer says, "I tried an experiment upon a frog with a few drops of this oil dissolved in ether, and placing the web of the foot under the microscope, found an almost entire suspension of the circulation. The frog was insensible for a much longer time than when the ether alone was used. He is now bright and ready for another experiment—to which I proceed:

"I exposed him to the vapor of a few drops of fusel oil dissolved in a dram of New England rum for six minutes, when he closed his eyelids and seemed under its influence. He was then placed upon the stand of the microscope, but there was no appearance of circulation found in any of the vessels of the web, and after my absence for half an hour I returned and found him dead."

Several queries suggest themselves:—

1st. Is there any fusel oil in sulphuric ether?

2d. Can the fusel oil be removed from the chloroform?

3d. Would the vapor of New England rum, rot-gut whisky, (which contains this oil,) produce anæsthetic effect?

4th. In what other liquors is this oil found?—*Boston Med. & Surgical Journal.*

***Recipe for Diseased Gums, Cleansing the Mouth, &c.***

| | | |
|---|---|---|
| 2 | gallons | spirits wine, 93 proof, in stone or glass demijohn. |
| 6 | drams | root pyretia. |
| 6 | " | star aniseed. |
| 8 | " | benzoni gum. |
| 4 | " | cloves. |
| 6 | " | cinnamon. |
| 2 | " | cochineal. |
| 4 | " | garllac. |

Powder all these well, the cochineal to a fine powder, put them in the spirit and shake well the mixture morning and evening, for fifteen days, expose to the sun sometime every day. Draw clear the liquor and add to it 4 drams essential oil minth, 4 drams cochlearia do., mix well three or four days more, and filter through white blotting paper in a glass funnel.—*Dental Recorder.*

## Figures and Tables

**Figure f1:**